# THE RELATIONSHIP BETWEEN ALLOSTATIC LOAD AND PROGRESS OF MULTIMORBIDITY AMONG OLDER AMERICANS

**DOI:** 10.1093/geroni/igad104.3654

**Published:** 2023-12-21

**Authors:** Rolla Saud Mira

**Affiliations:** King’s College London, London, England, United Kingdom

## Abstract

To examine the relationship between allostatic load and progress of multimorbidity over 10 years among older Americans and whether it mediates socioeconomic inequalities in multimorbidity.

Methods Six Waves of Health and Retirement Study (HRS), a longitudinal survey of older American adults from 2006 -2016. Multimorbidity was indicated by 5 chronic conditions: diabetes, heart conditions, lung diseases, cancer, and stroke. Socioeconomic factors were education, total wealth, poverty-income ratio (income), and race/ethnicity. Behavioural factors were smoking, excessive alcohol consumption, physical activity, and body mass index (BMI). Allostatic load was used as a biomarker of stress and was indicated by a combination of total cholesterol, high-density lipoprotein, glycosylated hemoglobin, c-reactive protein, waist circumference, and high blood pressure. Two Multilevel mixed effects generalized linear models were constructed to assess the association between allostatic load and progress of multimorbidity and whether allostatic load explained socioeconomic inequalities in the progress of multimorbidity. All variables included in the analysis were time-varying except gender, race/ethnicity, and education.

Results Allostatic load was significantly associated with the progress of multimorbidity even after adjusting for socioeconomic and behavioural factors with a rate ratio (RR) of 1.12 (95%CI: 1.11, 1.13). The association between socioeconomic factors and multimorbidity was slightly attenuated after adjusting for allostatic load. Socioeconomic factors were also associated with changes in allostatic load.

Conclusion Biological markers of stress indicated by allostatic load were associated with the progress of multimorbidity. Allostatic load appears to be induced by adverse socioeconomic factors and mediates the relationship between socioeconomic factors and progress of multimorbidity.

